# A Prospective Study Comparing Stapler and Open Surgical Technique of Hemorrhoidectomy

**DOI:** 10.7759/cureus.36304

**Published:** 2023-03-17

**Authors:** Mohal Kumar, Deepak Pankaj, Nitesh Kumar, K Abhishek, Vibhuti Bhushan, Yasir Tajdar, Pooja Kumari, Sweta Muni

**Affiliations:** 1 General Surgery, Indira Gandhi Institute of Medical Sciences, Patna, IND; 2 Microbiology, Indira Gandhi Institute of Medical Sciences, Patna, IND

**Keywords:** surgical procedures, stapler hemorrhoidectomy, recurrence, hemorrhoids, complications

## Abstract

Introduction

Hemorrhoids are basically anal cushions which turn out to be pathological giving rise to bleeding, pain and protrusion outside the anal canal. The chief complaint of patients with hemorrhoids is bleeding from the rectum which is usually painless and associated with episodes of defecation. The study was done to assess postoperative pain, time duration of the procedure, complications in the postoperative period, return to normal work, and recurrence between the stapler and open hemorrhoidectomy for grade III and IV hemorrhoids.

Material and methods

The present prospective study was conducted among 60 patients in the General Surgery department at Indira Gandhi Institute of Medical Sciences (IGIMS), Patna, Bihar over the duration of two years presenting with grade III and IV degree hemorrhoids. Thirty patients each were divided into open hemorrhoidectomy and stapled hemorrhoidectomy groups. The study evaluated variables like operative time, stay at the hospital and postoperative complications and compared them between the two techniques. Follow-up of patients was done at regular intervals. Evaluation of postoperative pain was done using visual analogue scale (VAS) with ranges from 0 to 10. We evaluated the data using the chi-square test with a p-value <0.05 as significant.

Results

Of 60 patients, 47 (78.3%) were males and 13 (21.7%) were females with a male: female ratio being 3.6:1. The operating time and hospital stay were much less in the stapler hemorrhoidectomy group as compared to the open procedure group. Also, postoperative pain (visual analogue scale) was less in the stapler hemorrhoidectomy group with 36.7% of patients presenting with pain at one week, 23.3% at one month and 3.3% at three months in the open hemorrhoidectomy group whereas 13.3% presenting as pain in one week, 10% presenting at one month and none presenting at three months in the stapler hemorrhoidectomy group. There was recurrence seen in 10% of cases at three months in the open hemorrhoidectomy group as compared to the stapler hemorrhoidectomy group where no recurrence was found at three months follow-up.

Conclusion

Hemorrhoid offers a variety of surgical modalities of treatment. We have come to the conclusion that stapled hemorrhoidectomy has less complications and good patient compliance. It can be an effective option in the treatment of third and fourth-grade hemorrhoids. With proper training and expertise, stapler hemorrhoidectomy is a better and reliable technique for hemorrhoid surgery.

## Introduction

Being one of the most frequent anorectal conditions, hemorrhoids are basically anal cushions which turn out to be pathological giving rise to bleeding, pain and protrusion outside the anal canal. They can affect around 30% to 40% of the general population sometimes or others during their lifetime. It is generally said that hemorrhoids are the price we humans pay for our erect or upright posture [1]. Hemorrhoids can both be external and internal. While internal hemorrhoids arise from the subepithelial plexus in the anal canal above the dentate line, external hemorrhoids are vascular plexuses present outside and covered with skin [2]. Internal hemorrhoids can be classified into four grades according to the degree of prolapse although the symptoms may not be conducive with the extent or severity of patients' sufferings [3]. Hemorrhoids may have varied clinical presentations such as bleeding, pain, mucus discharge, itching and something coming out of the rectum [4]. Bleeding from the rectum which is painless and associated with episodes of defecation, is the most common complaint by patients with hemorrhoids. The patient usually complains of dripping of blood in the toilet pan. Due to direct arteriovenous communication in the hemorrhoidal mass, the blood is typically bright red [5]. Internal hemorrhoids based on their appearance and degree of prolapse are graded into four types (Goligher’s classification): (1) Grade I: Bleeding present but no prolapse; (2) Grade II: Hemorrhoids prolapse outside the anal canal but reduce spontaneously; (3) Grade III: Hemorrhoids prolapse outside the anal canal and require manual reduction; and (4) Grade IV: Hemorrhoids prolapse all the time and are irreducible, it also includes acutely thrombosed hemorrhoids and those involving prolapse of rectal mucosa circumferentially [6]. Dietary and lifestyle modification, stool softeners, laxatives along with oral flavonoids and calcium dobesilate constitute medical management of hemorrhoids [7]. First and second-degree hemorrhoids are usually treated conservatively with lifestyle and dietary modifications and medications [8]. Various nonoperative treatment modalities also available include sclerotherapy, cryotherapy, rubber band ligation, infrared coagulation and radiofrequency ablation. However, hemorrhoidectomy remains the most common procedure performed for hemorrhoids and minimally invasive procedure for hemorrhoids (MIPH) which is basically a stapled haemorrhoidopexy is a safer and newer modality of treatment approach performed worldwide [9,10]. Dr. Antonio Longo in 1998 proposed this stapled procedure for hemorrhoidectomy known as circular stapled rectal mucosectomy with the aim to reduce the size of internal hemorrhoids by interrupting their blood supply with subsequent reduction of the size of the vascular anal cushions [11]. The present study was done to compare stapler hemorrhoidectomy and open hemorrhoidectomy in the management of third and fourth-degree hemorrhoids with their outcomes and complications during the postoperative period and follow-up.

## Materials and methods

The present study was prospective type and done among 60 patients in the General Surgery department at IGIMS, Patna, Bihar with a duration of two years. Those patients who presented with grade III and IV degree hemorrhoids on clinical examination and willing to give consent were included in the study. Patients with thrombosed piles and associated perianal conditions like abscess, anal fissure, rectal ulcer and rectal prolapse were excluded from the study. Approval from the institute’s ethical committee was taken before the start of the study (1600/IEC/2020/IGIMS) and informed consent was also taken from all patients involved in the study. The sampling technique for this study was based on nonprobability purposive sampling. Thirty patients each were divided into two groups as open hemorrhoidectomy and stapled hemorrhoidectomy groups. Surgery was carried out under spinal type of anaesthesia and patient positioning was done in lithotomy position for all the cases. Evaluation of postoperative pain was done utilizing visual analogue scale (VAS) ranging from 0 to 10 categorized into mild (0-3), moderate (4-7) and severe (8-10). Follow-up of patients was done at regular intervals at a period of one week, one month and three months. The interpretation of the data was performed using Microsoft Excel (Microsoft® Corp., Redmond, WA). The quantitative data obtained were expressed as percentage in tabular form and were evaluated using SPSS version 20 (IBM Corp., Armonk, NY) by chi-square test with p<0.05 as a significant value.

## Results

Sixty patients were part of this study, out of which 30 patients each were studied in both the open and stapler hemorrhoidectomy group. Of 60 patients, 47 (78.3%) were males and 13 (21.7%) were females with male: female ratio being 3.6:1. The maximum number of patients (55%) belonged to the age group 31 to 50 years, followed by age group 11 to 30 years (31.7%) and age group 51 to 70 years (13.3%) respectively. The operating time and hospital stay were much less in the stapler hemorrhoidectomy group as compared to the open procedure group. Also, postoperative pain (visual analogue scale) was less in the stapler hemorrhoidectomy group. The stapler group had an early return to normal activities as compared to the open hemorrhoidectomy group (Table 1).

**Table 1:** Clinical presentation of hemorrhoid patients

Characteristics		Total cases N=60	Stapler Haemorrhoidectomy N=30	Open Haemorrhoidectomy N=30	P-value
Age Group (years)	11-30	19	9/30 (30%)	10/30 (33.3%)	0.048
	31-50	33	16/30 (53.3%)	17/30 (56.7%)	
	51-70	8	5/30 (16.6%)	3/30 (10%)	
Operating time (in minutes)		-	24±6.2	46±10	0.0021
Hospital stay		-	1±1.2 (in days)	3±1.2 (in days)	0.001
Post-operative pain (visual analogue scale at 24 hours post-operative)		-	1.92±0.23	5.19±0.41	0.062
Return to activity (Normal routine Work)			4±1.2	14±3.4	0.042

Among the studied patients, the predominant symptom was bleeding per rectum in 73.3% of cases followed by mucus discharge in 63.3% of cases. The least common complaint was pain in 18.3% of cases. Other symptoms included perianal itching and protrusion out of the anal canal (Table 2).

**Table 2:** Symptoms of hemorrhoid patients

Symptoms	N=60 (%)
Bleeding	44 (73.3%)
Mucus discharge	38 (63.3%)
No discharge	22 (36.7%)
Perianal Itching	20 (33.3%)
Something protruding out of the anal canal	18 (30%)
Pain	11 (18.3%)

The patients were followed up for complications during postoperative period up to a period of three months. Pain was the symptom present in both the groups with 36.7% of patients complaining of pain in the open group while 13.3% of patients complained of pain in the stapled hemorrhoidectomy group. In the open hemorrhoidectomy group, pain was present in 36.7% of patients in one week postoperatively, 23.3% of patients in one month follow-up and only 3.3% in three months follow-up. In stapler hemorrhoidectomy, 13.3% of patients presented with pain in one week follow-up while 10% of patients presented with pain in one month follow-up while none presented with pain in three months follow-up. Bleeding was present in 6.7% of patients in the open hemorrhoidectomy group while none reported bleeding in the stapler group. One case of incontinence was present in the open group till one week follow-up. Recurrence was present in 13.3% of patients in the open hemorrhoidectomy group which persisted in 10% of patients till three months follow-up while one patient (3.3%) presented with recurrence in the stapled hemorrhoidectomy group after three months follow-up (Table 3).

**Table 3:** Incidence of various postoperative complications in open and stapler hemorrhoidectomy group

Complications	Open Hemorrhoidectomy Group n=30				Stapler Hemorrhoidectomy Group n=30			
	Total (post-operative till 3 months follow-up)	At 1 week (n=30)	At 1 month (n=30)	At 3 months (n=30)	Total (post-operative till 3 months follow-up)	At 1 week (n=30)	At 1 month (n=30)	At 3 months (n=30)
Pain	11 (36.7%)	11 (36.7%)	7 (23.3%)	1 (3.3%)	4 (13.3%)	4 (13.3%)	3 (10%)	0
Bleeding	2 (6.7%)	1 (3.3%)	1 (3.3%)	0	0	0	0	0
Incontinence	1 (3.3%)	1 (3.3%)	0	0	0	0	0	0
Recurrence	4 (13.3%)	0	1 (3.3%)	3 (10%)	1 (3.3%)	0	0	1 (3.3%)

## Discussion

Hemorrhoids are a common condition of the anorectal region and it presents as swelling and protrusion of the cushions of the anal canal affecting millions of people during their lifetime. Prolonged standing posture and constipation have been implicated as the main causative factors for this medical and socioeconomic problem [3]. The main clinical feature of hemorrhoid is rectal bleeding which is painless and occurs during bowel movement and is described by patients as fresh blood in a toilet bowl [12]. Other symptoms include mucus discharge, pain and something protruding out of the rectum. In cases with third and fourth-degree hemorrhoids, surgery is the procedure of choice where there are different kinds of procedures with their own pros and cons [13]. Surgical hemorrhoidal excision has its own set of complications which are stenosis of the anal canal, persistent discharge, incontinence which may be partial or complete, bleeding, and pain which sometimes can be severe and persistent. Even with the evolution of various newer techniques of hemorrhoid treatment like infrared coagulation, cryotherapy, laser hemorrhoidectomy, etc., the open hemorrhoidectomy technique remains the most commonly performed surgical procedure for the treatment of hemorrhoids [14]. Antonio Longo introduced “stapled hemorrhoidectomy” in 1998 at the World Endoscopic meeting in Rome which showed superior results and early recovery and return to work. This procedure was specifically designed to gain patients' satisfaction and due to less postoperative pain, this procedure has widely gained acceptance by most surgeons around the world [15]. The majority of patients were male in this present study with male to female ratio being 3.6:1. Both the procedures whether open or stapler techniques for hemorrhoids were not free of complications but the incidence of complications was found to be less in the patients operated with staplers. Pain was seen in more patients (36.7%) in the open hemorrhoidectomy group which decreased to 3.3% of patients in three months follow-up. Incidence of pain was less in the stapler group with 13.3% of cases at one week follow-up and no symptoms of pain at three months follow-up. Recurrence was also seen in the open hemorrhoidectomy group in 13.3% of cases as compared to 3.3% of cases in the stapler hemorrhoidectomy group. No occurrence of incontinence was found in the stapler hemorrhoidectomy group, whereas the occurrence of minor incontinence in 3.3% of cases during one week follow-up was found in the open hemorrhoidectomy group. The average operation time in the present study was less in stapler hemorrhoidectomy patients as compared to the open group. Almost all the studies done by various researchers from the past till present show similar results where the time taken for stapler hemorrhoidectomy is quite less [16-18]. Postoperative stay in the hospital was more in the open hemorrhoidectomy group as the wound becomes exposed with a larger raw area and required observation for any bleeding and also pain present due to the exposed sensitive anoderm area. Even patients returned to normal activity within four days of surgery in stapled hemorrhoidectomy group in the present study while returning to normal activity required almost two weeks in the open hemorrhoidectomy group. The postoperative hospital stay and return to normal routine work are also in agreement with studies done by other researchers (Table 4). So it was noticed that in the open hemorrhoidectomy group patients needed more time during surgery, more hospital stay and more time to return to normal activities.

**Table 4:** Average operative time, hospital stay and return to normal activity time in the stapler and open haemorrhoidectomy group done by various researchers.

Various studies	Operative time (mins)		Hospital stay (days)		Return to normal routine activity (days)	
	Stapler Haemorrhoidectomy	Open Haemorrhoidectomy	Stapler Haemorrhoidectomy	Open Haemorrhoidectomy	Stapler Haemorrhoidectomy	Open Haemorrhoidectomy
Present Study	24±6.2	46±10	1±1.2	3±1.2	4±1.2	14±3.4
Sachin and Muruganathan (2017) [16]	33	44	2	4	8	15
Singh et al. (2018) [1]	<30	<40	1	3	2-7	7-13
Malyadri and Allu (2021) [17]	40 (38-40)	50 (48-51)	1	3	3	5
Surati et al. (2022) [18]	34 (20-50)	40 (20-60)	1.5	2.4	3 (2-8)	20.5 (6-46)

## Conclusions

Hemorrhoids can be troublesome sometimes with intermittent bleeding and perianal discharge which can be a cause of concern to the patient. Hemorrhoid offers a variety of surgical modalities of treatment. We have come to the conclusion that stapled hemorrhoidectomy has less complications and good patient compliance. It can be an effective option in the treatment of third and fourth-grade hemorrhoids. Further clinical trials are also needed to prove the results of our study. It is also recommended that along with surgical management, patients should be advised of dietary and lifestyle modifications to prevent a recurrence. Further studies with more number of patients and a longer period of follow-up are recommended to attain more encouraging results.
